# Assessing Granger-Causality in the Southern Humboldt Current Ecosystem Using Cross-Spectral Methods

**DOI:** 10.3390/e22101071

**Published:** 2020-09-24

**Authors:** Javier E. Contreras-Reyes, Carola Hernández-Santoro

**Affiliations:** 1 Departamento de Estadística, Facultad de Ciencias, Universidad del Bío-Bío, Concepción 4081112, Chile; 2 División de Investigación Pesquera, Instituto de Fomento Pesquero, Valparaíso 2361827, Chile; carola.hernandez@ifop.cl

**Keywords:** granger-causality, cross-spectrum, spectral density, southern humboldt current ecosystem, anchovy

## Abstract

The southern Humboldt Current ecosystem is an important topic among researchers working on the drivers of pelagic species’ biological indicators. While sea surface temperature is believed to be a major driver in anchovies’ (*Engraulis ringens*) reproductive and body condition indicators, this paper shows that regional drivers such as Pacific decadal oscillation anomalies also influence these biological processes. In addition, a warm condition could trigger increased gonad development of anchovies and synchronization of body condition dynamics with local environmental conditions stemming from sea turbulence and Ekman transport. To test the statistical significance of causality between two time series and determine the direction of causality, the frequency-domain Granger-causality method is considered. Therefore, this study provides additional predictive information, derived from past data on anchovy reproductive and feeding activities. The study could be useful for researchers working on relationships of environmental conditions and pelagic species to predict biological processes’ maximum and minimum peak movements and anchovy abundance in the southern Humboldt Current ecosystem.

## 1. Introduction

The Earth’s climate systems have important implications for the ecology and physiology of key marine species. One of these systems is the 1997–98 El Niño–Southern Oscillation (ENSO) phenomenon, which impacts the small pelagic ecosystem in northern Chile [1]. The findings of Ulloa et al. [2] considered the importance of studying species’ dependent attributes for evaluating the biological impacts of environmental perturbations produced by the ENSO phenomenon. Specifically, a long-term climate variability estimate was produced in northern Chile [1], showing anomalies associated with El Niño events, which affected the abundance, recruitment, reproduction, adult biomass and environment of coastal pelagic fish such as the anchovy (*Engraulis ringens*) on different temporal and spatial scales [3].

In addition, large local-scale phenomena such as regime shifts, ENSO cycle (El Niño-La Niña), seasonality, coastal-trapped waves and upwelling events have been postulated as driving biological processes in northern Chile [3]. Examples include, (1) distribution of anchovy changes in space and depth during warm conditions such as El Niño; (2) after 2002 female length classes showing a positive trend of the gonadosomatic index (GSI), indicating favorable development of anchovy gonads coinciding with the period of prevailing cool conditions without strong, warm events (i.e., El Niño 1997–98); and (3) cool conditions will favor the presence of cold coastal water, a shallower thermocline, stronger upwelling and higher productivity [4]. Differences could be related to how anchovies synchronize reproductive dynamics with local environmental conditions to foster survival of offspring [5]. These dynamics are ultimately influenced by other long-term climatic processes, such as mild temperature changes [6]. This is due to warm/cool phase changes produced by environmental changes associated with sea surface temperature (SST) which affects upwelling habitat conditions. Thus, regional factors could reduce the horizontal upwelling habitat, leading to more anchovy abundance on the coast [4,5].

More recently, [7] characterized how the anchovy synchronizes reproductive dynamics. They investigated how local environmental variables such as the multivariate ENSO index (MEI) and Humboldt Current index (HCI) [8,9] couple with the reproductive timing of anchovy, measured by GSI. Contreras-Reyes et al. [7] concluded that the beginning and end of the anchovy spawning period fluctuate mainly because of environmental factors, that the environment is affecting the timing of gonad development of anchovies off northern Chile, that the strength of the relationship varied according to female body size and that this leads to at most two-monthly spawning events per year. However, the authors presented these relationships from an autocorrelation function perspective, which ignores the possibility that an environmental variable could cause the biological one. Distinguishing causality from correlation is a crucial problem in connected dynamic systems, especially when system variables appear positively coupled at certain times but negatively at others, depending on system state [10,11]. Additionally, studies are lacking on important aspects such as short- and long-term trends of local and regional environmental conditions, which influence the local and regional oceanographic conditions linked to climate change.

To test the statistical significance of causality between two time series and determine the direction of causality, the Granger-causality method [12] was employed in [13,14,15]. This approach motivated the present study, as a need has arisen to characterize the short- and long-term causality of the environment in anchovies’ biological processes. We applied the Granger-causality method to test whether statistically significant feedback among environmental indexes and GSI and condition factor (CF) [16] time series exists. In this case, HCI and regional environmental variability drivers such as Pacific decadal oscillation (PDO) and Antarctic decadal oscillation (AAO), plus local environmental variability drivers such as Ekman transport (ET), the sea turbulence index (TI) and SST can provide additional predictive information from the past for the anchovy’s reproductive and feeding activities. The Granger-causality method focuses on the type of dependence among the variables; the reproductive and feeding activities by interval of length with more dependence on environmental variables; and the expected variance produced in GSI monthly means and intra-annual behavior [17].

This paper is organized as follows: Granger-causality based on cross-spectrum methods is described in Section 2. Section 3 presents the data collected off northern Chile to illustrate the evidence for causality for local and regional environmental drivers and biological ones. Discussion concludes the paper in Section 4.

## 2. Frequency-Domain Granger-Causality Test

To analyze the cause–effect interaction between the variables, i.e., to identify the stimuli and responses for the variables along the time axis, the Granger-causality test [12] was carried out. Granger-causality is a feedback mechanism method based on cross-spectral methods. Let $x_{t}$ be a stationary process with no-periodic components, so the spectral representation of $x_{t}$ is

(1) $$x_{t}=\int_{-\pi }^{\pi }f_{x}\left(\lambda \right)e^{i\lambda t}d\lambda ,$$

where λ∈(−π,π) are the Fourier frequencies. $f_{x}\left(\lambda \right)$ is the spectral density of $x_{t}$ given by

(2) $$f_{x}\left(\lambda \right)=\frac{1}{\pi }\sum_{h=-\infty }^{\infty }\gamma_{x}\left(h\right)e^{-i\lambda h},$$

where $\gamma_{x}\left(h\right)=E\left[x_{t}x_{t+h}\right]$, h∈Z, is the autocovariance function (ACF) of $x_{t}$ and $i=\sqrt{-1}$. Given that for some processes the exact ACF is difficult to obtain, it is approximated by the sample ACF, ${\hat{\gamma }}_{x}\left(h\right)$, to then obtain the periodogram as estimator of the spectral density [18].

The main idea of Granger-causality is measuring the causality between two time series based on spectral representation of Equation (1). To this end, the cross-spectral density and the coherence function will be defined next.

**Definition** **1.** *Let $x_{t}$ and $y_{t}$ be two stationary time series, where $y_{t}$ has a spectral representation as in Equation (1) but with spectral density $f_{y}\left(\lambda \right)$, λ∈(−π,π), given in Equation (2) with autocovariance function $\gamma_{y}\left(h\right)=E\left[y_{t}y_{t+h}\right]$. Thus, the coherence function $C_{y\to x}\left(\lambda \right)$ between $x_{t}$ and $y_{t}$ is defined as*

$$C_{y\to x}\left(\lambda \right)=\frac{{\left|C\left(\lambda \right)\right|}^{2}}{f_{x}\left(\lambda \right)f_{y}\left(\lambda \right)},$$

*respectively, where $C\left(\lambda \right)=E[f_{x}\left(\lambda \right)\bar{f_{y}\left(\lambda \right)}]$ is the cross-spectral density between $x_{t}$ and $y_{t}$, $\bar{f_{y}\left(\lambda \right)}$ is the conjugate of the complex function $f_{y}\left(\lambda \right)$ and “y→x” denotes that $y_{t}$ Granger-causes $x_{t}$.*

The cross-spectrum density is the Fourier transformation of cross-covariance of two time series, which gives us the degree of relationship between two time series at different frequencies. The cross-spectral density of Definition 1 is a complex function of λ and the absolute value of the coherence function is a real function defined in [0,1] that measures the relationship degree between $x_{t}$ and $y_{t}$, given by the correlation coefficient square between the frequency components of the time series. If $C_{y\to x}\left(\lambda \right)=1$, $C\left(\lambda \right)=f_{x}\left(\lambda \right)f_{y}\left(\lambda \right)$, i.e., independence exists between spectral densities of both time series. Additionally, on the contrary, if $C_{y\to x}\left(\lambda \right)=0$, strong dependence exists between spectral densities of both time series.

Considering the univariate time series $x_{t}$ and $y_{t}$ of Definition 1, [19] showed that in a two-dimensional vector of time series, $z_{t}=\left(x_{t},y_{t}\right)$ at time t=1,…,n, where $z_{t}$ is a finite *p*-order vector autoregressive (VAR) process (see, e.g., [20]) of the form

(3) $$\Phi \left(B\right)z_{t}=\varepsilon_{t},\;t=1,\ldots ,n;$$

where $\Phi \left(B\right)=\sum_{i=0}^{p}\phi_{i}B^{i}$ is a 2×2 lag polynomial with $\phi_{0}=1$ and $B^{i}z_{t}=z_{t-i}$, and the error vector $\varepsilon_{t}$ is a white noise process with $E[\varepsilon_{t}]=0$ and $E[\varepsilon_{t}\varepsilon_{t}^{⊤}]=\sum$, where ∑ is a 2×2 positive definite variance-covariance matrix. The VAR process may include a constant, a trend or dummy variables.

Considering a Cholesky decomposition using a lower triangular matrix G, the ∑ matrix can be decomposed as $G^{⊤}G=\sum^{-1}$ such that $E[\eta_{t}\eta_{t}^{⊤}]=I$ and $\eta_{t}=G\varepsilon_{t}$. With the assumption that $z_{t}$ is a stationary process, the moving average (MA) representation of the process is

$$z_{t}=\Phi {\left(B\right)}^{-1}\varepsilon_{t}=\left(\begin{array}{cc} \phi_{11}{\left(B\right)}^{-1} & \phi_{12}{\left(B\right)}^{-1}\\ \phi_{21}{\left(B\right)}^{-1} & \phi_{22}{\left(B\right)}^{-1} \end{array}\right)\left(\begin{array}{c} \varepsilon_{1t}\\ \varepsilon_{2t} \end{array}\right)=\left(\begin{array}{cc} \psi_{11}\left(B\right) & \psi_{12}\left(B\right)\\ \psi_{21}\left(B\right) & \psi_{22}\left(B\right) \end{array}\right)\left(\begin{array}{c} \eta_{1t}\\ \eta_{2t} \end{array}\right)=\Psi \left(B\right)\eta_{t},$$

where $\Psi \left(B\right)=\Phi {\left(B\right)}^{-1}G^{-1}$. Then, the spectral density of $x_{t}$ is

(4) $$f_{x}\left(\lambda \right)=\frac{1}{2\pi }\left(|\psi_{11}\left(e^{-i\lambda }\right){|}^{2}+{\left|\psi_{12}\left(e^{-i\lambda }\right)\right|}^{2}\right).$$

**Definition** **2.** *[21] Considering the spectral density of Equation (4), the measure of causality of $y_{t}$ over $x_{t}$ is*

(5) $$M_{y\to x}\left(\lambda \right)=\log\left(1+\frac{2\pi f_{x}\left(\lambda \right)}{|\psi_{11}\left(e^{-i\lambda }\right){|}^{2}}\right)=\log\left(1+\frac{|\psi_{12}\left(e^{-i\lambda }\right){|}^{2}}{|\psi_{11}\left(e^{-i\lambda }\right){|}^{2}}\right).$$

The null hypothesis that $y_{t}$ does not Granger-cause $x_{t}$ and the alternative hypothesis that $y_{t}$ Granger-causes $x_{t}$ at frequency λ is then given by

(6) $$H_{0}:\;M_{y\to x}\left(\lambda \right)=0\;\mathrm{versus}\;H_{1}:\;M_{y\to x}\left(\lambda \right)\neq 0.$$

To obtain a suitable Wald statistic, $M_{y\to x}\left(\lambda \right)$ is obtained by replacing $|\psi_{11}\left(e^{-i\lambda }\right)|$ and $|\psi_{12}\left(e^{-i\lambda }\right)|$ in Equation (5) by the estimated values obtained from the fitted VAR [20]. However, as in [19], the disadvantage of Wald’s statistic is the exact computation of $|\psi_{12}\left(e^{-i\lambda }\right)|$. To solve this, the following facts should be considered:

(a) From Equation (5), if $|\psi_{12}\left(e^{-i\lambda }\right){|}^{2}=0$, then $M_{y\to x}\left(\lambda \right)=0$, which implies that $y_{t}$ does not Granger-cause *x* at frequency λ∈(−π,π);
(b) From Cholesky decomposition, we have $\Psi \left(B\right)=\Phi {\left(B\right)}^{-1}G^{-1}$ and
  $$\psi_{12}\left(B\right)=\frac{-g_{22}\phi_{12}\left(B\right)}{\det[\Phi (B)]},$$
  where $g_{22}$ is the lower-diagonal element of $G^{-1}$ and $G^{-1}$ is a positive matrix given that ∑ is a positive definite variance-covariance matrix.

From (a) and (b) and the Euler representation, we have that y↛x for a frequency λ∈(−π,π) if

(7) $$|\phi_{12}\left(e^{-i\lambda }\right)|=|\sum_{j=1}^{p}\phi_{12,j}(\cos(j\lambda )-i\sin(j\lambda ))|=0,$$

with $\phi_{12,j}$ the (1,2)th-element of $\phi_{j}$ defined in Equation (3), j=1,…,p. Given that sin(jλ)=0 if λ=0 or π, from Equation (7) we have that

(8) $$\sum_{j=1}^{p}\phi_{12,j}\cos\left(j\lambda \right)=\sum_{j=1}^{p}\phi_{12,j}\sin\left(j\lambda \right)=0.$$

Let $\alpha_{j}=\phi_{11,j}$, and $\beta_{j}=\phi_{12,j}$ and j=1,…,p; the process $x_{t}$ can be modeled by harmonic regression

(9) $$x_{t}=\sum_{j=1}^{p}\alpha_{i}x_{t-i}+\sum_{j=1}^{p}\beta_{i}y_{t-i}+\varepsilon_{1t}.$$

If *i* exists such that $\beta_{i}\neq 0$, then $y_{t}$ Granger-causes $x_{t}$; if $\beta_{i}=0$ for all *i*, $y_{t}$ does not Granger-cause $x_{t}$. This implies the mean square error (MSE) of $E[x_{t}|y_{t},y_{t-1},\ldots ]$ is smaller than $E[x_{t}|x_{t},x_{t-1},\ldots ]$. Then, the null hypothesis of Equation (6) is equivalent to $H_{0}:\;R\left(\lambda \right)\beta^{⊤}=0$, where R(λ), λ∈(0,π), is a 2×p matrix defined by

(10) $$R\left(\lambda \right)=\left(\begin{array}{cccc} \cos(\lambda ) & \cos(\lambda ) & \ldots & \cos(p\lambda )\\ \sin(\lambda ) & \sin(2\lambda ) & \ldots & \sin(p\lambda ) \end{array}\right)$$

and $\beta =(\beta_{1},\ldots ,\beta_{p})$.

Under null hypothesis, $y_{t}$ is Granger-cause of $x_{t}$ if $\beta_{i}\neq 0$ for a specific *i* versus the alternative hypothesis, $y_{t}$ does not Granger-cause $x_{t}$ if $\beta_{i}=0$, for all *i*. Therefore, the null hypothesis is tested with a joint Fisher *F*-test of the harmonic regression model given in Equation (9). The *F* statistic is approximately Fisher-distributed as F(2,n−2p) for a frequency λ∈(0,π). A critical value for rejecting the null hypothesis is 0.05 if a 95% confidence level is considered.

The proposed test developed by [19] postulates that a variable $y_{t}$ affects another variable $x_{t}$ at a finite time horizon. Typically, some environmental and biological systems are modeled as cointegrated (non-stationary) ones [13]; thus, the definition of causality at frequency zero is equivalent to the concept of long-run causality. For stationary systems, it is assumed that $x_{t}$ is predicted using only the past of the series. If the spectral density of the resulting forecast error at low frequencies can be explained by the additional past information of $y_{t}$, then $y_{t}$ is said to be a long-run cause for $x_{t}$. Both cases must be considered in marine ecosystem modeling.

## 3. Application to Southern Humboldt Current Ecosystem

The anchovy is the main small pelagic fishery species in the upwelling southern Humboldt Current ecosystem (SHCE), with stock in southern Peru and northern Chile ($16^{{}^{\circ}}$–$24^{{}^{\circ}}$ S). In this system, the PDO has been described as the main driver of pelagic species alternation in several upwelling systems on eastern coasts, such as California’s and the Humboldt Current [1,9]. The PDO oscillates between warm and cool phases associated with sardine/anchovy dominance. Specifically, the PDO presented a warm phase from 1989 to 2000, and then a cool phase started that is predicted to last until 2025. Anchovies’ existence on northern Chile’s coasts is related to local environmental conditions, characterized by high-intensity coastal upwelling processes in summer due to the intensity of southern winds [22]. The coastal upwelling processes generate strong temperature gradients coupled with the phytoplankton community, with the coolest and warmest habitats inside and outside the coast, respectively, and abundant anchovy biomass in hot zones.

In this section, we analyze the influence and Granger-causality of regional (HCI, PDO and AAO) and local (IT, ET and SST) factors in anchovy reproductive and body condition indicators (GSI and CF, respectively) in the upwelling Humboldt Current system in northern Chile.

### 3.1. Data and Software

**Figure 1:**
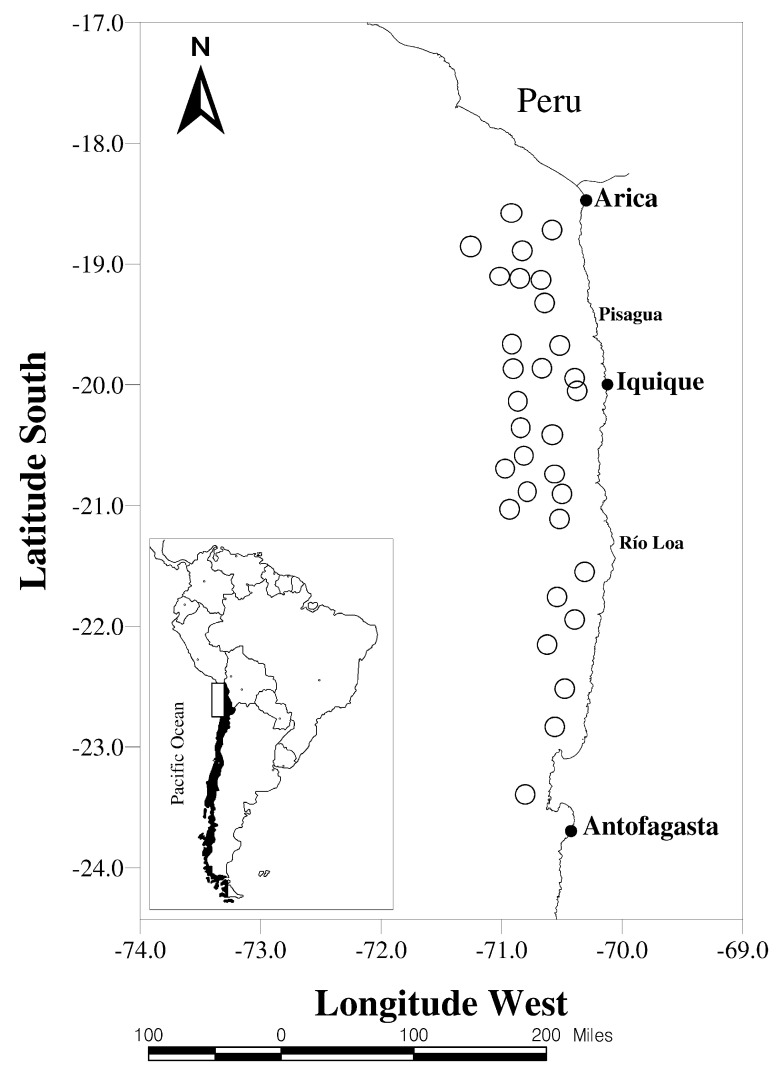
Study area restricted to anchovy landings in the north of Chile ($18^{{}^{\circ}}$21${}^{\prime }$–$24^{{}^{\circ}}$00${}^{\prime }$ S). The circles represent gravity centers of capture distributions of anchovies in northern Chile during the study period (1989:01–2016:12). Source: Contreras-Reyes et al. [7].

The study area was restricted to anchovy landings in northern Chile ($18^{{}^{\circ}}$21${}^{\prime }$–$24^{{}^{\circ}}$00${}^{\prime }$ S), along the Peru–Chile maritime border at the port of Antofagasta ($23^{{}^{\circ}}$26${}^{\prime }$ S; see Figure 1).

#### 3.1.1. Environmental Data

On a local scale, the 1989–2017 monthly means of meteorological and oceanographical station records of Antofagasta port ($23^{{}^{\circ}}$26${}^{\prime }$ S) were analyzed for SST, ET and TI. SST data correspond to images of global SST level 4 and are produced daily in a grid of $0.25^{{}^{\circ}}$ by NOAA’s National Climatic Data Center with a spatial resolution of 25 km obtained from the Group for High Resolution SST. The satellite information on SST was analyzed in [4] with the help of daily images by constructing 1896 interpolated weekly images using empirical orthogonal functions (DINEOF) to complete the missing data between 1981 and 2017 [4]. For each interpolated weekly image, the average SST was extracted for the first 10 nautical miles from the coast. SST, air temperature, chlorophyll, mean sea level and wind magnitude and direction were variables used to estimate ET and TI, according to methods proposed by [23,24], respectively.

At the regional scale, three indexes were used: the HCI (http://www.bluewater.cl/HCI/), containing atmospheric–oceanographic activity along the Chilean coast [8]; the PDO (http://research.jisao.washington.edu/pdo, University of Washington) and the monthly AAO anomalies (http://www.cpc.noaa.gov, NOAA-Climate Prediction Center).

#### 3.1.2. Biological Data

The study period was from 1989 to 2017 and the studied small pelagic species were part of the Chile–Peru shared stock, located between $16^{{}^{\circ}}$ S and $24^{{}^{\circ}}$ S. Analyzed biological information came from the Fisheries Development Institute’s (IFOP) monitoring program and was financed by the Chilean Undersecretariat of Fisheries and Aquaculture (SUBPESCA). Data were generated from biological sampling at landings or aboard fishing vessels. A total of 25,000 samplings are available on average per year; 50 specimens are selected for each sampling. Sex, total weight and gonad weight (0.01 g) were determined (visual inspection according to [25]).

Reproductive condition was determined by calculating the GSI [4,7] at month *t* as the percentage of gonad monthly mean weight ($G_{t}$) in relation to monthly mean body weight ($W_{t}$) for all fish sampled as

(11) $$GSI_{t}=100\frac{G_{t}}{W_{t}-G_{t}}\;\left(\%\right).$$

Fulton’s condition factor (CF) [26] of all fish sampled was calculated at month *t* as follows:

(12) $$CF_{t}=100\frac{W_{t}}{L_{t}^{3}}\;\left(\%\right),$$

where $L_{t}$ is the monthly mean weight. In the denominator of Equation (12) exponent 3 is imposed as an approximation of the estimated exponent in the regression model given in [16].

#### 3.1.3. Software and Computational Implementation

All estimations and computational implementations were carried out with R software [27].

To evaluate the presence of unit roots in time series, the augmented Dickey–Fuller (ADF) [28] test was considered. For the presence of seasonal unit roots in time series, the Hylleberg–Engle– Granger–Yoo (HEGY) test [29] was considered. The ADF test was implemented in the adf.test function of the tseries package and the HEGY test was implemented in the hegy.test function of the uroot package. To detect significant frequencies in time series, the robust G-test was considered based on robust regression [30] and implemented in the robust.spectrum function of the GeneCycle package.

The estimation of cross-spectral density and coherence function was implemented in the crossSpectrum function of IRISSeismic package. The Granger-causality test described in Section 2 was implemented in grangertest function of lmtest package. The VAR parameter estimation and frequency-domain-based Granger-causality test were respectively implemented in VAR and causality functions of vars package.

### 3.2. Results

**Figure 2:**
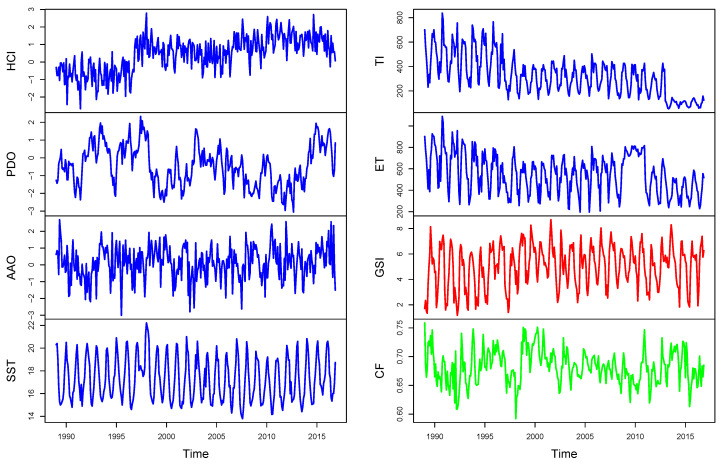
Time-series of the Humboldt Current index (HCI), Pacific decadal oscillation (PDO), Antarctic decadal oscillation (AAO), sea surface temperature (SST), sea turbulence index (TI) and Ekman transport (ET), for 1989:01–2016:12.

Figure 2 plots the environmental and biological variables described. The plot of the regional environmental drivers seems to be non-stationary, though it could well be trend-stationary. Additionally, the plot of local environmental and biological indicators looks stationary with an annual cyclical pattern. Some relationships can be highlighted between indicators, mainly produced by the 1996–1998 El Niño phenomenon, which can be crucial for analysis of causality (see Section 2). Considered time series include the most relevant events (significant trend breaks identified in [7]): the first in 1995:5, the second and highest in 1998:6 and the last one in 2002:3 [31].

We started with unit root tests to verify if the time series are stationary I(0) (and/or I(1)). Table 1 shows, based on the ADF unit root tests, that the null hypothesis of a non-stationary unit root was rejected for all variables, except HCI and TI (at 95% confidence level), and ET (at 98% confidence level). Next, the ADF test was applied to the detrended processes but gave a rejected null hypothesis of a non-stationary unit root. As mentioned in Section 3.1.2, local environmental and biological time series presented significant frequencies at 6 and 12 months, as confirmed by the p-values of the robust G-test. The HEGY test did not present seasonal unit roots but confirmed the results of the ADF test for HCI and ET. Therefore, p-values smaller than 0.01 are not shown in Table 1, where p-values of GSI and CF also suggest seasonal stationarity. Therefore, the differentiation $\left(1-B\right)y_{t}=y_{t}-y_{t-1}$ was only imposed for HCI, ET and TI time series for the next analysis.

**Figure 3:**
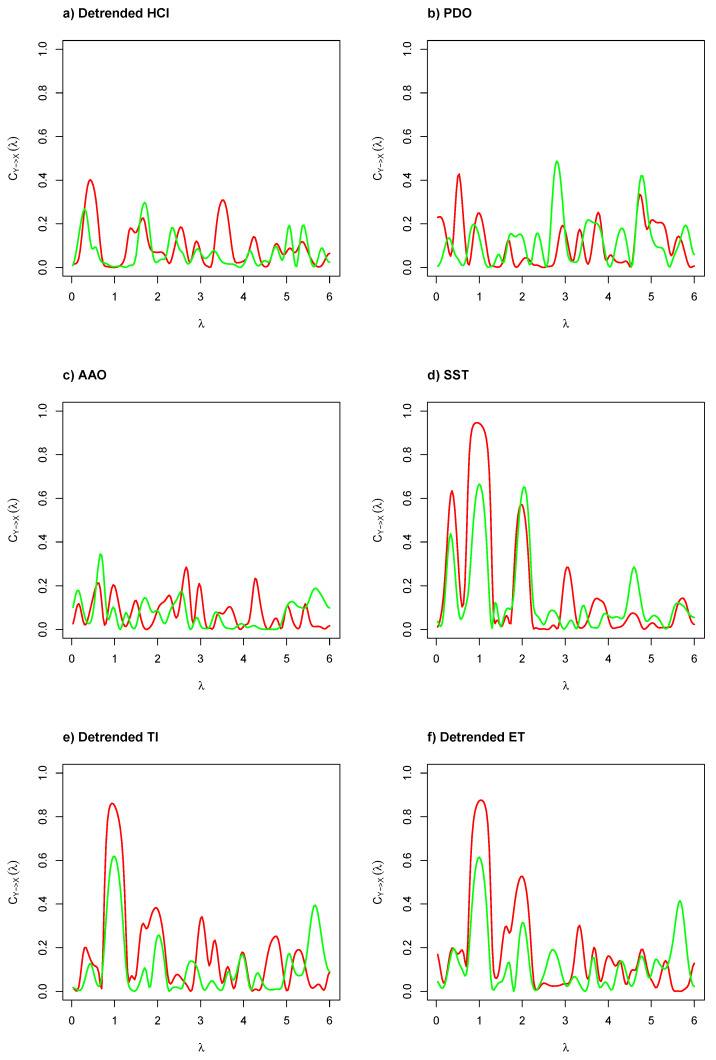
Coherence function between a regional or local environmental ($y_{t}$) and biological ($x_{t}$) indicator, where $x_{t}$ represents the gonadosomatic index (GSI; solid red line) or condition factor (CF; solid green line).

Figure 3 illustrates the coherence functions by frequency based on cross-spectral density between environmental and biological time series. We can probably consider a linear relationship between two tested time series’ frequencies if the coherence function is large at specific frequencies. As can be seen in Figure 3a–c, the coherence function was close to 0 for frequencies in (0,2π) and the highest coherence was obtained at λ≈0.52 (12 months or annual cycle), and the second one was obtained at λ≈1.05 (6 months or inter-annual cycle), obtained by seasonal components of GSI and CF. However, in Figure 3d–f, the coherence function is close to 1 for frequencies in (0,π) and close to 0 for frequencies in [π,2π), where the highest coherence was obtained at λ≈1.05. This means that evidence exists of causality at different lags (but related to low number of predictors) based on the cross-spectrum analysis when compared the local environmental and biological time series, mainly produced by the significant frequency detected by robust G-test.

**Figure 4:**
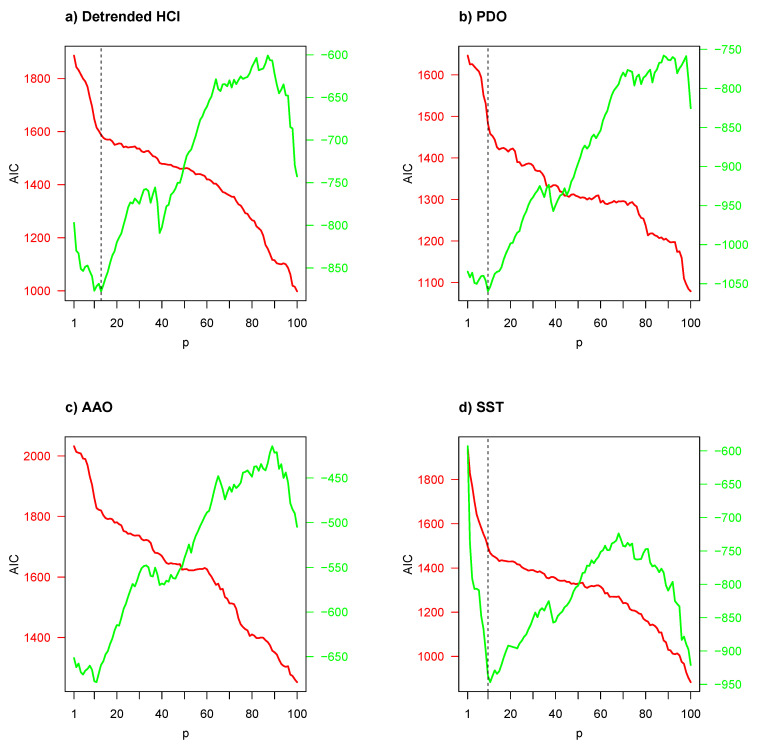

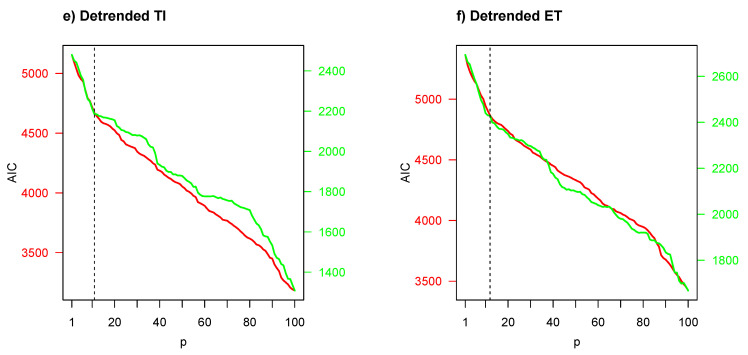
Akaike’s information criteria (AIC) versus the number of predictors/regressors (*p*) for VAR models considering regional or local environmental and biological (GSI (solid red line) or CF (solid green line)) time series.

Subsequently VAR models were evaluated for bivariate time series, composed from the local/regional environmental and biological time series. Figure 4 illustrates Akaike’s information criterion (AIC) with respect to number of predictors/regressors (*p*). The AIC was used to determine the number of predictors/regressors, where the smallest AIC values indicate the “best” models. Given that the Granger-causality test is very dependent and highly influenced by the selection of predictors/regressors, an appropriate *p* for the objective variable $z_{t}$ in Equation (3) should be determined by AIC. No environmental indicator had the minimum AIC value when the GSI was considered as a biological indicator; thus in the first instance a cut-off point (or marked reflection point, vertical dotted line) was considered. However, when the CF was considered as a biological indicator, a minimum AIC value emerged among p=10 and p=13 predictors in panels (a)–(d), but for panels (e) and (f), the same situation as in the GSI case occurred.

According to AIC results of Figure 4, a VAR model with a low number of predictors was considered for frequency-domain Granger-causality test in the first instance, and a high number of predictors (p=100) in the second instance, except for bivariate time series composed of CF and detrended HCI, PDO, AAO and SST. The results of these frequency-domain Granger-causality tests are presented in Table 2. For the low *p* and GSI as *X* case, the null hypothesis that *Y* does not Granger-cause *X* was rejected at a 95% confidence level only for PDO, SST and detrended TI as *Y* cases, but the null hypothesis could not be rejected for a high *p*. This means that evidence exists of PDO, SST and detrended TI Granger-causing GSI. For the CF case, only for SST and detrended ET was the null hypothesis rejected with a 95% confidence level for a low *p*, but again, the null hypothesis could not be rejected for a high *p*, proving that SST and detrended ET Granger-cause CF. This corroborates the evidence of Granger-causality at different lags in the cross-spectrum analysis when compared to the local environmental and biological time series.

Contreras-Reyes et al. [7] detected cross-correlation between detrended HCI and GSI; however, we observed in Table 2 evidence that Granger-causality running from detrended HCI to biological processes is weak for no-coupling. Moreover, the weakest causal effect holds for AAO according to tests of both biological processes. This occurs because the Antarctic dynamic system is not synchronized with the biological processes of small pelagics from the Chile-Peru shared stock. Therefore, AAO could be discarded as a strong signal in biological indicator estimates and cannot be used to detect the presence of external drivers that might be unknown in the modeling.

## 4. Conclusions and Discussion

The SHCE is an important topic among researchers working on the drivers of pelagic species’ biological indicators. However, the selection of “correct” drivers for identifying causality in the SHCE can be difficult. Sometimes the variables are positively coupled, but at other times they appear unrelated or even negatively coupled depending on the local/regional environmental indicator [4]. Chile–Peru shared stock exhibit radically different dynamic control regimes by large local-scale phenomena, such as regime shifts, ENSO cycle, seasonality, coastal-trapped waves and upwelling events, causing the correlations between small pelagic species and phytoplankton and producing a change sign [5].

Given the importance of the issue of climate change, this study revisited the question of whether environmental factors influence reproductive and body conditions of the anchovy by using a frequency-domain Granger-causality test. This technique can capture nonlinearities, potentially intrinsic to data generating processes for local/regional environmental and biological processes, for instance, due to the structural breaks explained in [4,7]. These structural breaks in SST, GSI and CF were determined by the 1996–1998 El Niño event, and this study presented evidence that a regional indicator such as PDO could be an important factor in anchovy development. Moreover, given that the Granger-causality test is highly influenced by the number of predictors/regressors *p* in the VAR model, for a high *p* (∼100), this implies that a variable $y_{t}$ affects another variable $x_{t}$ at a infinite time horizon; however, this concept is not addressed by the frequency-domain Granger-causality test, as is postulated in the conclusions of [19]. Therefore, our study presented the evidence of Granger-causality for a low *p* (10≤p≤13, see Table 2) based on a cut-off point criterion. In addition, the study highlighted that PDO, SST, TI and ET have always been important in predicting reproductive and body condition activity, as researchers working on links between environmental conditions and pelagic species can predict movements of the maximum and minimum peaks of biological indicators. This study could also be useful for predicting anchovy abundance in the SHCE [5,11].

While SST is believed to be a major cause of GSI and CF time series [3,4], there is a debate that suggests that regional drivers such as PDO anomalies also drive these biological indicators. However, the evidence in terms of the latter line of reasoning is mixed. Hernández-Santoro et al. [4] highlighted that seasonal change of SST explained and caused GSI and CF, determining a delay of the start and maximum GSI, and a negative relationship with CF. In addition, [4] showed a gradual SST increase mainly during the austral winter starting in 2006, due to a phase change in the PDO [32]. Therefore, this study corroborates that a warm condition could trigger a rise in anchovy gonad development, so the GSI could be explained by SST as local environmental indicator. Additionally, anchovy synchronize their body condition dynamics with the local environmental conditions given by TI and ET.

This study is based on previous cross-correlation analysis of [4,7], where the the question of causality in a dynamic ocean SHCE was addressed with a different methodology. To answer that question, the Granger-causality concept provides predictability, rather than correlation of these studies, giving more evidence of causation between time-series variables [11], and filling the gap of determining Granger-causation over correlation. Although correlation is neither necessary nor sufficient to establish causation, it remains deeply ingrained in our heuristic thinking [10]. For example, detrended HCI does not Granger-cause GSI and/or CF, but they are correlated. On the other hand, lack of correlation does not imply lack of Granger-causation. However, Granger-causality addresses prediction rather than correlation as the criterion for causation in time series and assumes that causes can be separated from effects. This is possible in purely stochastic system; however, it is not defined for all systems, such as deterministic dynamic systems. Additionally, while we only analyzed Granger-causality in addition to the correlation analysis by [4,7], we must highlight that GSI and CF were used as partial proxies for reproductive and body condition factors of anchovy, respectively. Thus our evidence of causality between the processes should not be associated with a real correlation between two variables. To confront this issue, a detrended cross-correlation analysis [33] will be required, which was beyond the scope of this study.

Finally, further work must consider a spatial-temporal approach for causality [34]. Neglecting these issues could also lead to spurious research outcomes, ignoring more significant influences local/regional environmental drivers have on biological ones. However, our objective was to obtain the first evidence of causality at a space-point scale but over a long period.

## Figures and Tables

**Table 1:** Statistics and p-values (in parenthesis) for augmented Dickey–Fuller (ADF) and Hylleberg– Engle–Granger–Yoo (HEGY) tests; *p*-values of robust G-test (RG) are also presented for frequencies related to 6 and 12 months. Other frequencies are not presented, given that *p*-values were higher than 0.05 (no significant frequencies).

Variable	Hypothesis	ADF	RG	HEGY
HCI	$s_{6}$	-	0.605	-
	$s_{12}$	-	0.754	-
	I(0)	−3.226 (0.083)	-	-
	I(1)	−13.192 (0.010)	-	-
	$t_{1}$	-	-	−2.207 (0.462)
PDO	$s_{6}$	-	0.602	-
	$s_{12}$	-	0.049	-
	I(0)	−5.088 (0.010)	-	-
AAO	$s_{6}$	-	0.682	-
	$s_{12}$	-	0.744	-
	I(0)	−7.639 (0.010)	-	-
SST	$s_{6}$	-	0	-
	$s_{12}$	-	0	-
	I(0)	−4.999 (0.010)	-	-
TI	$s_{6}$	-	0.041	-
	$s_{12}$	-	0	-
	I(0)	−2.919 (0.189)	-	-
	I(1)	−19.462 (0.010)	-	-
ET	$s_{6}$	-	0	-
	$s_{12}$	-	0	-
	I(0)	−3.780 (0.020)	-	-
	I(1)	−20.727 (0.010)	-	-
	$t_{1}$	-	-	−3.165 (0.084)
GSI	s=6	-	0	-
	s=12	-	0	-
	I(0)	−7.555 (0.010)	-	-
CF	s=6	-	0.011	-
	s=12	-	0	-
	I(0)	−5.386 (0.010)	-	-

**Table 2:** Frequency-domain Granger-causality test results in the Y→X sense for two VAR models: the first and second ones considered $p_{1}$ (low) and $p_{2}$ (high) numbers of predictors, respectively. For each model, the tests included the Fisher statistic (*F*) and *p*-value.

*Y*	*X*	$p_{1}$	*F*	*p*-Value	$p_{2}$	*F*	*p*-Value
Detrended HCI	GSI	13	1.353	0.177	100	0.741	0.930
	CF	13	0.905	0.548	100	-	-
PDO	GSI	10	2.075	0.025	100	0.852	0.786
	CF	10	1.831	0.052	100	-	-
AAO	GSI	10	0.514	0.881	100	0.358	1.000
	CF	10	0.878	0.553	100	-	-
SST	GSI	10	9.158	<0.01	100	1.258	0.152
	CF	10	2.357	0.001	100	-	-
Detrended TI	GSI	11	1.935	0.033	100	1.210	0.175
	CF	11	1.607	0.092	100	0.946	0.608
Detrended ET	GSI	12	0.842	0.608	100	0.647	0.984
	CF	12	2.191	0.011	100	0.897	0.704
